# Exclusive Use of Air as Gas Tamponade in Rhegmatogenous Retinal Detachment

**DOI:** 10.1155/2017/1341948

**Published:** 2017-07-13

**Authors:** Kang Yeun Pak, Seok Jae Lee, Han Jo Kwon, Sung Who Park, Ik Soo Byon, Ji Eun Lee

**Affiliations:** ^1^Department of Ophthalmology, Haeundae Paik Hospital, Inje University, Busan, Republic of Korea; ^2^Department of Ophthalmology, School of Medicine, Pusan National University, Busan, Republic of Korea; ^3^Medical Research Institute, Pusan National University Hospital, Busan, Republic of Korea; ^4^Research Institute for Convergence of Biomedical Science and Technology, Pusan National University Yangsan Hospital, Yangsan, Republic of Korea

## Abstract

**Purpose:**

To investigate outcomes of vitrectomy for rhegmatogenous retinal detachment (RRD) using air exclusively as the gas tamponade.

**Methods:**

This retrospective, interventional, consecutive case series involved reviewing medical records of patients that underwent vitrectomy and gas tamponade for RRD between January 2013 and December 2015. Patients whose eyes were treated exclusively with air tamponade since July 2014 were assigned to the air group, while those treated with heterogeneous gas agents before June 2014 were assigned to the control group. The primary outcome was the primary reattachment rate. Best-corrected visual acuity (BCVA) and duration to detect redetachments were assigned as the secondary outcomes.

**Results:**

The air group and the control group included 71 and 72 eyes, respectively. The primary reattachment rate was 94.4% in the air group and there was no significant difference with 94.4% in the control group (*p* = 0.951). BCVA was significantly better in the air group at 1 month (*p* = 0.021) but not at 3 months postoperatively (*p* = 0.561). Redetachments were recognized earlier in the air group (9.3 ± 0.5 days) compared with those in the control group (21.3 ± 7.4 days) (*p* = 0.041).

**Conclusions:**

In cases of simple RRD with sufficient removal of subretinal fluid, air could be considered for use as gas tamponade. This trial is registered with KCT0002358.

## 1. Introduction

Pars plana vitrectomy (PPV) with gas tamponade is widely popular for the treatment of rhegmatogenous retinal detachment (RRD). Nonexpansile gases, such as 18% sulfur hexafluoride (SF6), 14% octafluoropropane (C3F8), and room air, are commonly used as gas tamponade at the end of vitrectomy [1, 2]. Of these, 18% SF6 and 14% C3F8 last for at least 1 month and as such are named long-acting gases, whereas room air has a much shorter half-life of only 1 day [3]. Generally, the success rate using long-acting gas as tamponade had been thought to be higher than that using room air in RRD [4].

Due to technical advances, the success rate of vitrectomy for RRD has soared; however, surgeons consider the success rate as well as patient compliance. Recently, a prospective study of inferior RRD by Zhou et al. [5] showed that the results with room air tamponade were not inferior to those with long-acting gas, which agrees with our own retrospective data [6]. On the basis of these results, since July 2014, we have used room air exclusively as gas tamponade at the end of vitrectomy for RRD.

Herein, we review our case results with room air tamponade and compare them with previous results using heterogeneous gas tamponade.

## 2. Methods

The current study was a retrospective, interventional, consecutive case series. The medical records of consecutive patients that underwent vitrectomy and gas tamponade for primary RRD between January 2013 and December 2015 were reviewed. We excluded cases treated by scleral buckling, vitrectomy combined with scleral buckling, or use of silicone oil tamponade. Patients who had a history of intraocular surgery, except for cataract and uveitis or retinal vascular disorders, were excluded. The institutional review board of Pusan National University Hospital approved the study protocol, and the protocol complied with the tenets of the Declaration of Helsinki.

### 2.1. Choice of Surgical Procedure

Scleral buckling and vitrectomy combined with scleral encircling were usually used in young phakic RRD and severe proliferative vitreoretinopathy cases, respectively. Silicone oil tamponade after vitrectomy was applied in cases with failure to sufficiently remove subretinal fluid or the vitreoretinal proliferative membrane. We deemed vitrectomy with gas tamponade as the best choice in cases other than those described above.

### 2.2. Surgical Procedures

All patients were operated on by a single surgeon (SW Park), who used the Constellation (Alcon Laboratories Inc., Fort Worth, TX) sutureless 23-gauge (G) or 25 G vitrectomy system and a noncontact wide viewing system, the Resight 700 (Carl Zeiss Meditec AG, Jena, Germany). Phacoemulsification was combined concurrently at the surgeon's discretion. If necessary, to confirm the presence of posterior vitreous detachment or epiretinal membrane, triamcinolone acetonide was applied during the PPV. For shaving the peripheral vitreous, an assistant usually indented the sclera. Prophylactic laser photocoagulation was applied only around retinal breaks or lesions predisposed to retinal detachment, not on the normal-looking retina. During vitrectomy, perfluorocarbon liquid (PFCL) was used if necessary. Patients were instructed to maintain a face-down position for 1 to 3 days after the operation.

### 2.3. Data Collection

The patients were divided into two groups: the air group and the control group. The air group underwent vitrectomy after July 2014 and received only air tamponade. The control group underwent vitrectomy between January 2013 and June 2014 and received heterogeneous gas tamponade.

The following baseline characteristics were collected: type of gas, age, sex, intraocular pressure (IOP), lens status, axial length, high myopia, involvement of macula, number of retinal breaks, range of retinal detachment, presence of inferior breaks, use of PFCL during operation, and best-corrected visual acuity (BCVA), which was quantified using lines of Snellen visual acuity. The inferior breaks were defined as one or more breaks inside the retinal detachment located between the 4 and 8 o'clock positions.

The primary reattachment was defined as a retina reattached at 3 months after a single operation without any additional procedure. The primary reattachment rate was compared between the two groups. BCVA was compared at 1 and 3 months postoperative. IOP measurements at 1 and 7 days postoperative were analyzed. In the redetached cases, duration to detect the redetached retina, total number of surgeries, final BCVA, and presence of inferior breaks was investigated and compared.

### 2.4. Statistical Analysis

All statistical analyses were performed using SPSS for Windows 21.0 (SPSS Inc., Chicago, IL). BCVA was converted to a logarithm of the minimum angle of resolution (logMAR) for statistical analysis. Differences between the two groups were assessed using an independent Student's *t*-test (continuous factors) or chi-square test (categorical factors). In the subgroup of redetached cases, differences between the two groups were assessed using the Mann–Whitney *U* test or Fisher's exact test. *p* values <0.05 were considered statistically significant.

## 3. Results

### 3.1. Baseline Characteristics

The air group comprised 174 eyes in patients with primary RRD who underwent surgery for RRD over an 18-month period beginning in July 2014. Of these 174 eyes, the following were excluded: 51 eyes (29.3%) that received a scleral buckling procedure, 29 eyes (16.7%) that received a combined scleral buckling with vitrectomy, and 23 eyes (13.2%) that were treated with silicone oil as tamponade. Finally, a total of 71 eyes (40.8%) were included in the air group. The control group comprised 180 eyes in patients with primary RRD patients who underwent surgery for RRD for an 18-month period beginning in January 2013. Of these 180 eyes, the following were excluded: 64 eyes (35.6%) that received a scleral buckling procedure, 30 eyes (16.7%) that received a combined scleral buckling with vitrectomy, and 14 eyes (7.8%) that were treated with silicone oil as tamponade. Finally, a total of 72 eyes (40.0%) were included in the control group (Table 1). The air group comprised 34 men and 37 women with a mean age of 58.6 ± 8.8 years, while the control group comprised 40 men and 32 women with a mean age of 56.9 ± 9.8 years. In the air group, air tamponade was used exclusively. In the control group, air was used in 15 eyes, diluted SF6 in 55 eyes, and diluted C3F8 in two eyes. The baseline characteristics of each group are summarized in Table 2. There were no significant differences in terms of age, sex, IOP, lens status, axial length, proportion of high myopia, involvement of macula, number of retinal breaks, range of retinal detachment, presence of inferior breaks, use of PFCL, and BCVA.

### 3.2. Surgical and Visual Outcomes

The primary reattachment was observed in 67 of 71 eyes (94.4%) in the air group and 68 of 72 eyes (94.4%) in the control group, with no significant difference between the groups (*p* = 0.951, chi-square test).

In the air group, BCVA improved from 1.11 ± 1.05 logMAR (median, 20/125) at baseline to 0.23 ± 0.25 (median, 20/32) at 1 month and 0.18 ± 0.19 (median, 20/25) at 3 months postoperatively. In the control group, BCVA improved from 1.38 ± 1.11 logMAR (median, 20/300) at baseline to 0.33 ± 0.34 (median, 20/40) at 1 month and 0.20 ± 0.24 (median, 20/25) at 3 months. At 1 month postoperatively, BCVA was significantly better in the air group (*p* = 0.021), but not at 3 months (*p* = 0.561). The IOP of the air group changed from 13.0 ± 3.1 mmHg at baseline to 10.5 ± 3.1 at 1 day and 15.1 ± 4.2 at 7 days postoperatively. In the control group, IOP changed from 13.6 ± 2.7 mmHg to 17.2 ± 7.2 at 1 day and 15.6 ± 5.9 at 7 days. The IOP of the control group was significantly higher at 1 day (*p* < 0.001) but not at 7 days (*p* = 0.414) postoperatively. The postoperative clinical outcomes are summarized in Table 3.

In the subgroup of redetached retina cases, surgical failure was recognized significantly earlier in the air group (9.3 ± 0.5 days) compared with that in the control group (21.3 ± 7.4 days) (*p* = 0.041). The final reattachment was achieved in all cases, requiring 2.3 ± 0.5 and 2.5 ± 0.6 surgeries in total in the air group and control group, respectively. The final BCVA and presence of inferior breaks were not statistically different between the two groups (*p* = 0.556 and *p* = 0.465, resp.) (Table 4).

## 4. Discussion

In selected RRD cases where air was used as gas tamponade, noninferior results to long-acting gas have been reported [5, 7–10]. To the best of our knowledge, the current study is the first report of exclusive use of air as gas tamponade in vitrectomy for RRD. Our results revealed that air tamponade was noninferior to the heterogeneous gas as tamponade in RRD and had advantages in terms of earlier visual recovery and earlier detection of redetached retinas.

Tamponades serve as a barrier to prevent the movement of fluid between the vitreous cavity and the subretinal space. Once adhesion between the retina and retinal pigment epithelium (RPE) has been established, the barrier is no longer needed. Theoretically, retina-RPE adhesion occurs within 24 hours in situations without subretinal fluid (SRF) [11]. However, remnant SRF around tears can disturb the adhesion, in which cases, the use of tamponade should be extended until the SRF is absorbed. If tears are located in the superior retina, gravity keeps the tears isolated from SRF and use of a long-acting tamponade might be excessive. On the other hand, if tears are located in the inferior retina and viscous SRF remains, a short-acting gas such as room air might be inappropriate as tamponade. Several reports support this theory. Tan et al. [4] said that room air was comparable to long-acting gas not in RRD with inferior breaks but in those with superior breaks. They suggested that air tamponade should be used in cases involving the superior quadrants. Martínez-Castillo et al. conducted three important studies of vitrectomy for RRD with inferior breaks [8–10]. Their results suggested that RRD with inferior breaks could be treated well with vitrectomy even when using air tamponade [9] and that sufficient SRF drainage was an important factor in such cases [10].

The results of primary vitrectomy for RRD were first reported by Escoffery et al. [12]. Air was used as tamponade because no other tamponade was available at that time, and the success rate was 79%. Since then, treatment advances including the use of long-acting gases have led to higher success rates, and these gases have been considered superior to air as tamponade for RRD [4]. Recently, new technologies such as perfluorocarbon liquids, intraocular diathermy, wide-angle viewing systems, and advanced vitreous cutters have contributed to the sufficient removal of SRF and vitreous. Recently, success rates have become over 95% [6, 10, 13, 14], and air tamponades have been reappraised in RRD [5, 6, 10]. The authors retrospectively analyzed 206 cases of RRD using vitrectomy in which the success rate of air tamponade (36 eyes, 100%) was not inferior to that of the others (170 eyes, 95.5%) [6]. A recent randomized comparative prospective trial showed that air was not inferior to diluted C3F8 in RRDs with inferior breaks [5]. But their study designs, procedures, and results seem not to be well controlled; a randomization was failed especially in terms of high myopia. Their method of gas dilution was not general, and although success rates were comparable between the two groups (diluted C3F8: 78.1% and air: 84.4%), those were not comparable to those of other recent reports, 95% or over [6, 10, 13, 14].

Air tamponade has several advantages over long-acting gases, which require additional buying, keeping, and dilution, and it can reduce the cost and surgical time. Dilution and keeping these gases can lead to complications such as elevated IOP, faster absorption than expected, and toxicity due to gas contamination. Additionally, the results of the current study showed that visual recovery and recognition of surgical failure were faster using air. Although we found no significant difference in the final visual outcomes between the groups among the redetached retina cases, an earlier recognition of surgical failure would be expected to offer better outcomes [15, 16].

This study had several limitations. First, there were selective biases due to the retrospective nature. The current study did not include all types of RRD. This study enrolled only simple cases. The cases with insufficient removal of SRF and vitreoretinal proliferative membrane were filled with silicone oil and excluded. The cases treated with scleral buckling or encircling were also excluded. Second, air was compared with heterogeneous gases rather than long-acting gases because we could not find a control group with comparable baseline characteristics who received a long-acting gas. Third, there was a time gap between the surgeries in the two groups, during which technical advances or surgical skill might have had an impact. However, we reasoned that 18 months was too short a time to have a large effect. Finally, while a 3-month follow-up was too short to determine visual outcome, it was enough to evaluate anatomical outcomes and helped to reduce selective biases. In spite of these limitations, the baseline characteristics were quite comparable and a wide spectrum of RRD cases was consecutively enrolled. The current results should be interpreted in consideration of these limitations, and large randomized prospective studies will be needed in the future.

## 5. Conclusion

Air used exclusively as gas tamponade in vitrectomy for RRD in a consecutive case series spanning 18 months delivered anatomical and visual outcomes that were comparable to those of the control group receiving heterogeneous gases and also to those described in recent reports [10, 13, 14]. Early visual recovery and early recognition of surgical failure were advantages of air tamponade. In cases of vitrectomy for simple RRD with sufficient removal of SRF, air tamponade can be considered as a substitute for a long-acting gas. A prospective, head-to-head, large-scale comparative study will be necessary in the future to validate the efficacy of air tamponade in RRD.

## Figures and Tables

**Table 1:** Choice of surgical method in each 18-month period.

Surgical methods	Before July 2014 (*n*, %)	After July 2014 (*n*, %)	*p* value
Vitrectomy + gas tamponade	72 (40.0)	71 (40.8)	0.914
Scleral buckling	64 (35.6)	51 (29.3)	0.214
Scleral buckling with vitrectomy	30 (16.7)	29 (16.7)	1.000
Vitrectomy + silicone oil tamponade	14 (7.8)	23 (13.2)	0.118
Total	180 (100)	174 (100)	

**Table 2:** Baseline characteristics of each group.

	Control group	Air group	*p* value
Type of gas (*n*) Air : SF_6_ : C_3_F_8_	15 : 55 : 2	71 : 0 : 0	<0.001^†^
Age (mean ± SD, yr)	56.9 ± 9.8	58.6 ± 8.8	0.351^∗^
Sex (M : F)	40 : 32	34 : 37	0.401^†^
BCVA (mean ± SD, logMAR)	1.38 ± 1.11	1.11 ± 1.05	0.143^∗^
IOP (mean ± SD, mmHg)	13.6 ± 2.7	13.0 ± 3.1	0.237^∗^
Axial length (mean ± SD, mm)	25.14 ± 2.11	24.83 ± 1.65	0.336^∗^
Proportion of high myopia (*n*, %)	23 (31.9%)	16 (22.5%)	0.282^†^
Macula On : off	32 : 40	36 : 35	0.614^†^
Number of retinal breaks (mean ± SD)	1.8 ± 1.3	1.6 ± 1.0	0.315^∗^
Range of retinal detachment (mean ± SD, hours)	4.4 ± 1.7	4.6 ± 1.9	0.357^∗^
Lens state Phakic : pseudophakic	58 : 14	52 : 19	0.422^†^
Inferior break (*n*, %)	28 (38.9%)	18 (25.4%)	0.111^†^
Use of PFCL (*n*, %)	20 (27.8%)	21 (29.6%)	0.336^†^

^∗^Independent student's *t*-test. ^†^Chi-square test. BCVA: best-corrected visual acuity; IOP: intraocular pressure; SD: standard deviation; logMar: logarithm of the minimum angle of resolution; PFCL: perfluorocarbon liquid.

**Table 3:** Clinical outcomes of each group.

	Control group	Air group	*p* value
Primary reattachment rate (*n*, %)	68/72 (94.4%)	67/71 (94.4%)	0.951^†^
Final reattachment rate (*n*, %)	72/72 (100.0%)	71/71 (100.0%)	1.000^†^
BCVA (mean ± SD, logMAR)			
At 1 month	0.33 ± 0.34	0.23 ± 0.25	0.021^∗^
At 3 months	0.20 ± 0.24	0.18 ± 0.19	0.561^∗^
IOP (mean ± SD, mmHg)			
At 1 day	17.2 ± 7.2	10.5 ± 3.1	<0.001^∗^
At 7 days	15.6 ± 5.9	15.1 ± 4.2	0.414^∗^

^∗^Independent student's *t*-test. ^†^Chi-square test. BCVA: best-corrected visual acuity; IOP: intraocular pressure; logMAR: logarithm of the minimum angle of resolution.

**Table 4:** Subgroup analysis of the eyes with redetached retinas.

	Control group	Air group	*p* value
Duration to detect the redetached (days)	21.25 ± 7.44	9.25 ± 0.50	0.041^∗^
Total number of surgery	2.25 ± 0.50	2.50 ± 0.58	0.686^†^
Final BCVA (mean ± SD, logMAR)	0.29 ± 0.28	0.22 ± 0.46	0.556^∗^
Inferior break (*n*, %)	2 (50%)	1 (25%)	0.465^†^

^∗^Independent student's *t*-test. ^†^Chi-square test. BCVA: best-corrected visual acuity; SD: standard deviation; logMAR: logarithm of the minimum angle of resolution.

## References

[1] Hakin K. N., Lavin M. J., Leaver P. K. (1993). Primary vitrectomy for rhegmatogenous retinal detachment. *Graefe’s Archive for Clinical and Experimental Ophthalmology*.

[2] Chan C. K., Lin S. G., Nuthi A. S., Salib D. M. (2008). Pneumatic retinopexy for the repair of retinal detachments: a comprehensive review (1986–2007). *Survey of Ophthalmology*.

[3] Thompson J. T. (1989). Kinetics of intraocular gases: disappearance of air, sulfur hexafluoride, and perfluoropropane after pars plana vitrectomy. *Archives of Ophthalmology*.

[4] Tan H. S., Oberstein S. Y. L., Mura M., Bijl H. M. (2013). Air versus gas tamponade in retinal detachment surgery. *British Journal of Ophthalmology*.

[5] Zhou C., Qiu Q., Zheng Z. (2015). Air versus gas tamponade in rhegmatogenous retinal detachment with inferior breaks after 23-gauge pars plana vitrectomy: a prospective, randomized comparative interventional study. *Retina*.

[6] Lee S. J., Kwon H. J., Park K. Y., Park S. W., Byon I. S., Lee J. E. (2016). Prognostic factors of anatomical success in microincisional vitrectomy for rhegmatogenous retinal detachment. *Journal of the Korean Ophthalmological Society*.

[7] Hotta K., Sugitani A., Uchino Y. (2004). Pars plana vitrectomy without long-acting gas tamponade for primary rhegmatogenous retinal detachment. *Ophthalmologica*.

[8] Martínez-Castillo V., Boixadera A., Verdugo A., García-Arumí J. (2005). Pars plana vitrectomy alone for the management of inferior breaks in pseudophakic retinal detachment without facedown position. *Ophthalmology*.

[9] Martínez-Castillo V., Verdugo A., Boixadera A., García-Arumí J., Corcóstegui B. (2005). Management of inferior breaks in pseudophakic rhegmatogenous retinal detachment with pars plana vitrectomy and air. *Archives of Ophthalmology*.

[10] Martínez-Castillo V. J., García-Arumí J., Boixadera A. (2016). Pars plana vitrectomy alone for the management of pseudophakic rhegmatogenous retinal detachment with only inferior breaks. *Ophthalmology*.

[11] Folk J. C., Sneed S. R., Folberg R., Coonan P., Pulido J. S. (1989). Early retinal adhesion from laser photocoagulation. *Ophthalmology*.

[12] Escoffery R. F., Olk R. J., Grand M. G., Boniuk I. (1985). Vitrectomy without scleral buckling for primary rhegmatogenous retinal detachment. *American Journal of Ophthalmology*.

[13] Bourla D. H., Bor E., Axer-Siegel R., Mimouni K., Weinberger D. (2010). Outcomes and complications of rhegmatogenous retinal detachment repair with selective sutureless 25-gauge pars plana vitrectomy. *American Journal of Ophthalmology*.

[14] Park S. W., Kwon H. J., Kim H. Y., Byon I. S., Lee J. E., Oum B. S. (2015). Comparison of scleral buckling and vitrectomy using wide angle viewing system for rhegmatogenous retinal detachment in patients older than 35 years. *BMC Ophthalmology*.

[15] Bonnet M. (1984). Clinical factors predisposing to massive proliferative vitreoretinopathy in rhegmatogenous retinal detachment. *Ophthalmologica*.

[16] Tseng W., Cortez R. T., Ramirez G., Stinnett S., Jaffe G. J. (2004). Prevalence and risk factors for proliferative vitreoretinopathy in eyes with rhegmatogenous retinal detachment but no previous vitreoretinal surgery. *American Journal of Ophthalmology*.

